# One step closer to an experimental infection system for Hepatitis B Virus? --- the identification of sodium taurocholate cotransporting peptide as a viral receptor

**DOI:** 10.1186/2045-3701-3-2

**Published:** 2013-01-11

**Authors:** Pei-Jer Chen, T-C Wu

**Affiliations:** 1 Hepatitis Research Center, National Taiwan University and Hospital, Hsinchu City, Taiwan; 2 Department of Pathology, Johns Hopkins University, Baltimore, USA

## Abstract

Following the successful cloning of receptor for SARS coronavirus a few years ago, Dr. Wenhui Li and colleagues raised attention again by publishing a possible receptor for hepatitis B virus in *eLife*. We will briefly review the significance of this finding and the future prospects of hepatitis B research.

## 

Among the five hepatotropic hepatitis viruses, only hepatitis B virus (HBV) and its satellite hepatitis D virus (HDV) still wait for the development of an in vitro infection system in cell culture. One hepatocellular carcinoma (HCC) cell line, HepaRG, can be infected at a modest efficiency after weeks of culture and induced differentiation [1]. Even primary human hepatocytes rapidly lose the capacity for HBV infection after brief cell culture. The HBV infection demands both intracellular and cell-surface factors. The intracellular requirements appear less stringent, as after transfection of HBV DNA into many HCC cell lines or mouse liver, which cannot be infected naturally, the viral genome is expressed and replicates actively. Thus, the failure of HBV infection is considered largely to be due to strict restriction on the interaction between HBV virions and the cell membrane.

The molecules on the cell membrane needed for HBV infection can be divided into two classes: low affinity and high affinity molecules. Among others, the heparan sulfates in the membrane proteins mediate the broad, but less specific, virus-cell interaction. However, the high affinity membrane partners for HBV remain elusive (the carboxypeptidase D found for duck hepatitis B virus may be the only serious contender [2]).

HBV envelope protein, namely the surface antigens, plays an essential role in the infection process. Both genetic and functional examination identified one domain in the N-terminus of HBV preS1 (amino acids 1–47) necessary for infection. This domain has been shown to function as a direct mediator for HBV by binding presumably cellular corresponding receptor(s) [3]. More importantly, the myristoylated peptide is shown to effectively block HBV infection in primary human hepatocytes and in the human hepatocyte-chimera mouse at a nanomolar concentration [4]. In fact, a clinical trial testing the efficacy of this peptide in preventing HBV infection has been ongoing [5]. Clearly, this preS1 peptide can be a useful probe to pull out the interacting cellular factors, including specific viral receptors.

Yan et al. have taken a reasonable approach to fish out possible HBV receptor(s) [6]. They engineered the first 2–47 amino acid peptide from PreS1 to increase its capacity to be cross-linked with proteins interacting with the cell membrane, without affecting its binding specificity. In order to obtain sufficient materials after cross-linking, they adopted the Tupaia hepatocytes, instead of human hepatocytes, for the experiments. The strategies actually brought down many membrane proteins, but in comparison with the negative control (homologous peptide without specific binding), they identified one cellular protein, NTCP (sodium taurocholate cotransporting peptide) by LC/MS/MS. The same protein was pulled down from human hepatocytes as well. The authors further produced HCC cell lines stably expressing NTCP and subsequently infected them with HBV or HDV. Immunofluorescence staining clearly demonstrated the expression of HBV and HDV proteins in these cell lines, suggestive of a successful viral infection. In addition, they documented a 2-4-fold increase of viral RNA and DNA after infection in the cell line by real-time PCR. They also showed a Southern blot supporting the presence of HBV covalently closed circular DNA in the infected cell, a well-recognized marker for productive HBV infection. Finally, they identified a stretch of 10 amino acids in the NTCP transmembrane domain, as the motif directly interacting with the PreS1 peptide.

NTCP is a transmembrane protein, usually located in the lateral surface (canalicular) of hepatocytes, which mediates bile acid transport [7]. Geographically, it is a good candidate for an HBV receptor. Moreover, the authors could convert the cell lines previously non-permissible to HBV infection to permissible by over-expression of NTCP, again supporting its possible role in the HBV infection process. This can be a critical and long-awaited discovery toward understanding HBV receptors and establishing an experimental HBV infection system.

Looking forward, we need to understand how NTCP interacts with both HBV envelope proteins and with other cellular proteins, especially through the motif embedded in the cell membrane. NTCP itself is not sufficient to allow HBV infection, as the majority of HepaRG cells were found to express NPCT but not to be infected [8]. NTCP might initiate or mediate molecular interactions that can overcome the cell-surface restrictions for viral entry. Such cooperative cellular or viral factors have to be discovered and demonstrated to enhance the efficiency of viral infection, at a level comparable to a natural one (hundreds or thousands fold viral amplification). For example, the authors can use the NTCP-expressing cell lines as the starting materials to systemically identify other factors (maybe carboxypeptidase D) and make these cell lines more productive and permissive to HBV infection. In the near future, standard virological assays for HBV infections, including Northern or Western blots, are expected to demonstrate the successful HBV infections in vitro.

The HBV research community has searched for HBV receptors for decades. Many candidates have been discovered and then discarded. The current study, however, took advantage of a well-documented viral peptide required for HBV entry in combination with a state-of-the-art proteomics platform. As a Chinese proverb says “a thousand-mile journey starts from one incremental step”. As such, the identification of NTCP as a potential viral receptor for HBV may serve as an important initial step for this journey, leading to the development of an HBV infection system to facilitate the HBV research and hepatitis B treatment.
